# Notes from L’Union Medicale

**Published:** 1869-04

**Authors:** 


Notes from L'Union Medicale.—Dr. Fort in a surgical memorandum remarks that among the numerous causes of facial neuralgia is one which authors do not notice, but which frequently occurs. This cause, almost certain to be a source of error to practitioners, is a lesion of the buccal mucous membrane, behind the molars, and produced by the evolution of a wisdom tooth. The functional disturbances brought about by this slight lesion are so intense as to lead sometimes to suspicion of more serious trouble.

M. Forget in a criticism of M. Fort's remarks, says it is very true that the evolution of wisdom teeth produces peculiar disturbances; and that the fact has already been pointed out by others. In a memoir published in 1828, on the various deviations of which the lower wisdom teeth are susceptible, Dr. Toirac, says M. Forget, reports six cases bearing on this point. In one of these cases the patient was subject to slight attacks of inflammation for a year after the left lower wisdom tooth began to appear. His cheek, not very much swollen, was extremely sensitive to the slightest pressure, while deglutition was almost impossible. The left tonsil was swollen, and the soft palate was very red. The posterior third of the crown of a wisdom tooth was found to be covered with a fleshy band, consisting of the gum, which was of a violet color, painful, and slightly ulcerated.

A worse case was reported by Dr. Desirabode, in 1851. A man of 25 years committed suicide, and it was supposed on account of violent dental neuralgia. At the autopsy it was found that the left lower wisdom tooth was directed horizontally from before backwards, the roots being in opposition with the base of the ramus, and its crown applied to the posterior molar, upon which it exerted strong pressure. The gum was greatly swollen. No other lesion existed in the dental apparatus.

M. Forget cites a still more aggravated case. A man 26 years old had been for a long time affected with neuralgia referred to the alveoli of the molar teeth on the right side of the lower jaw. The entire ramus was tumefied, and to a considerable degree. Impeded articulation; swelling of the whole *masseter* region, so to say; hyperostosis of the coronoid process. M. Maisonneuve, having exposed the bony tumor, applied the trephine in search of the tooth. The result not being satisfactory, resection of the jaw was done, at the alveolus of the first molar, and the condyle was disarticulated. The bone having been divided by a section parallel to its axis, M. Forget found several purulent cavities which had burrowed into its substance. It was an instance of medullary osteitis of the ramus of the jaw extending to the interior of the condyle, which was hollowed out, by a little purulent cyst opening close to the articular cartilage. The severe symptoms and the structural lesions had for their point of departure the abnormal enlargement of the wisdom tooth, which was shut up in the base of the coronoid process, and rising scarcely to the height of a millimetre above the level of the alveolus which it had hollowed out for itself. The tooth was of twice the normal size, the crown of it abutting against the neck of the adjoining tooth, in such a manner that in order to take rank in the dental arch, it would have been under the necessity of displacing from below upwards the molar which opposed its upward growth. It was this obstacle which compelled it to develop in the interior of the bone.

We drew off the above cases under the impression that it might possibly help the general practitioner, now and then, out of an obscure diagnosis, to be apprized of them. Since doing so, we received the communication—published in today's issue—entitled "Disease of the Jaws," and containing an account of a case similar to some of the foregoing.

